# Mitigation of Metabolic Diseases Through Personalized Nutrition: A Critical In‐Depth Review

**DOI:** 10.1002/fsn3.71387

**Published:** 2026-01-19

**Authors:** Muhammad Tayyab Arshad, M. K. M. Ali, Farhang Hameed Awlqadr, Sammra Maqsood, Ali Ikram, Md. Sakhawot Hossain, Muhammed Adem Abdullahi, M. M. Rashed

**Affiliations:** ^1^ Functional Food and Nutrition Program, Center of Excellence in Functional Foods and Gastronomy, Faculty of Agro‐Industry Prince of Songkla University Songkhla Thailand; ^2^ Department of Physics, College of Sciences Imam Mohammad Ibn Saud Islamic University (IMSIU) Riyadh Saudi Arabia; ^3^ Food Science and Quality Control, Halabja Technical College Sulaimani Polytechnic University Sulaymaniyah Iraq; ^4^ National Institute of Food Science and Technology University of Agriculture Faisalabad Faisalabad Pakistan; ^5^ University Institute of Food Science and Technology The University of Lahore Lahore Pakistan; ^6^ Department of Nutrition and Food Engineering Daffodil International University Dhaka Bangladesh; ^7^ Department of Food Science and Postharvest Technology Jimma University College of Agriculture and Veterinary Medicine, Jimma University Jimma Ethiopia

**Keywords:** dietary intervention, gut microbiome nutrigenomics, machine learning, nutrigenomics

## Abstract

Obesity, cardiovascular disease, and type 2 diabetes mellitus (T2DM) represent major global health and economic concerns. Traditional dietary recommendations frequently overlook individual heterogeneity in metabolic health. Personalized nutrition will provide a more focused approach to preventing chronic diseases by tailoring dietary recommendations according to lifestyle, metabolic, and genetic factors. This review examines the role of personalized nutrition in preventing metabolic diseases, with a focus on key components of nutrient‐gene interactions, including nutrigenomics, nutrigenetics, the gut microbiome, and biomarker‐based therapies. The main aim of this article is to investigate how variation within the microbiome and among genes impacts nutrient metabolism and make a case for successful evidence of individualized dietary intervention for obesity, diabetes, and cardiovascular disease. Future advancements in artificial intelligence, and genetic testing may make personalized nutrition more accessible, but there are questions about the price, feasibility, and ethics of its widespread use. The scope for personalized nutrition is wide and has strong potential to impact preventative health. An independent assessment calls for sustained scientific research, equitable accessibility, and ethical considerations that can make public health policies clinically relevant.

## Graphical abstract

The main objective of this review is to explore the impact of variation within the microbiome and among genes on nutrient metabolism and argue in favor of successful evidence of individualized dietary intervention for cardiovascular disease, diabetes, and obesity.

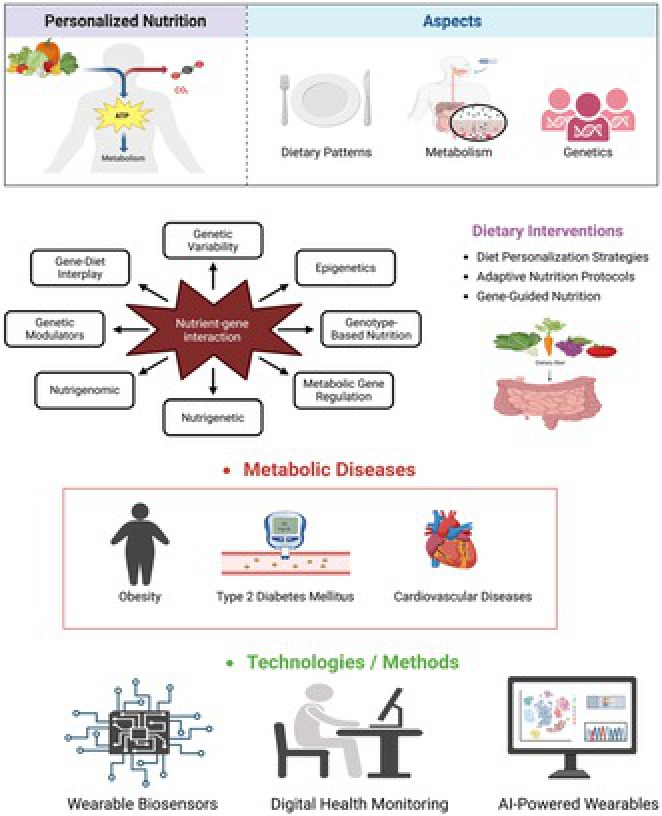

## 1 Introduction

Metabolic diseases mostly associated with Conditions include T2DM, obesity, insulin resistance, dyslipidemia, and excess body fat. All these conditions are considered a group of chronic disorders caused by anomalies in metabolic processes (Barrea et al. 2020; Shuvo et al. 2023). So massive is the level of exposure to metabolic diseases that it has become a global epidemic. For example, the World Health Organization reports that obesity prevalence has risen more than threefold since 1975, with over 650 million people being classified as obese in 2016 (Renzo et al. 2019; WHO 2017). Similarly, the prevalence of T2DM has increased, involving more than 537 million adults worldwide in 2021, and is projected to rise further to 783 million by 2045 (Khatir et al. 2024).

The increasing incidence of metabolic disorders is a significant concern for healthcare systems worldwide, particularly in terms of resource allocation and budgeting (Li et al. 2025). Recent data indicate that the prevalence of obesity among adults in the United States reached 40.3% in 2023, with higher rates in adults aged 40–59 years (46.4%) compared to those aged 20–39 years (35.5%) and ≥ 60 years (38.9%). Moreover, obesity‐related diseases are estimated to cost the U.S. healthcare system approximately $173 billion annually. Especially T2DM imposes a substantial economic and health burden. According to the IDF Diabetes Atlas, the global prevalence of diabetes among adults aged 20–79 years was 10.5% (536.6 million people) in 2021 and is projected to rise to 12.2% (783.2 million) by 2045. Global diabetes‐related health expenditures were estimated at USD 966 billion in 2021 and are expected to exceed USD 1054 billion by 2045 (Sun et al. 2022). The primary cause of global mortality is cardiovascular diseases, which represent a very symptomatic manifestation of metabolic disturbance and cause one‐third of all deaths (Zeinalian et al. 2022).

Metabolic disorders have huge societal and personal implications that go far beyond mere monetary consequences (Zhang et al. 2025). Diseases both reduce life expectancy and cause a sharp decline in quality of life, along with an increased risk of disability, psychological suffering, and decreased productivity (Hecker 2024). The rise in the incidence of metabolic illnesses worldwide is further contributing to the increase in health inequality gaps as they disproportionately affect lower‐income communities because of their restricted access to chances for physical activity, wholesome food, and healthcare (Chen and Chen 2022).

Despite their success in limiting the prevalence of many chronic diseases, traditional nutritional approaches have been criticized for failing to address the complexity of metabolic disorders. A key limitation of these approaches is that they provide generalized “one‐size‐fits‐all” dietary recommendations, which do not account for individual differences in genetics, metabolism, age, sex, or lifestyle (Renzo et al. 2019). A global dietary strategy is hard to design that would work uniformly for all as human nutritional needs may vary greatly from one individual to another based on his genetic profile, age, sex, metabolic status, and lifestyle (Barrea et al. 2020).

In developing nations, nutrient‐rich foods are scarce and unaffordable; consequently, diets are very high in unhealthy fats and refined carbohydrates, increasing the risk of metabolic disorders (Chen and Chen 2022). While other recommendations have helped raise public awareness and support disease prevention through diet, most do not provide the individualized guidance needed to effectively manage metabolic diseases (Bashiardes et al. 2018).

Metabolic disorders are complex, and therefore, nutritional recommendations should be individualized, taking into account a person's genetic predispositions, microbiome composition, and lifestyle factors (Kolodziejczyk et al. 2019). The research area of tailored nutrition has evolved as a practical strategy for the prevention and control of metabolic diseases due to the inadequacies of general dietary guidelines. Personalized nutrition is tailoring dietary advice to each individual's specific genetic, metabolic, and behavioral factors to achieve optimal health benefits and minimize the possibility of chronic diseases (van Ommen et al. 2017). This approach is informed by the fact that different people view various food products differently and that such customized treatments, as opposed to generalized dietary advice, could lead to the better management and prevention of metabolic diseases (Bubnov et al. 2015). With all those facts, the only thing that can be denied is that customized nutrition holds immense power to fully revolutionize the management and prevention of diseases. Personalized nutrition has the potential to better health outcomes and reduce the rate of metabolic diseases with high‐tailored dietary recommendations based on an individual's unique genetic, metabolic, and lifestyle characteristics (Barrea et al. 2020).

The main objective of this comprehensive review is to explore the impact of variation within the microbiome and among genes on nutrient metabolism and argue in favor of successful evidence of individualized dietary intervention for cardiovascular disease, diabetes, and obesity.

## 2 Key Components of Personalized Nutrition

### 2.1 Nutrigenomics

Nutrigenomics is the study of how nutrition, genes, and health interact. This field has been interested in how genes are expressed because of specific nutrients and how genetic variants can affect the response of someone to those nutrients (Li et al. 2022b). It's critical to the concept of personalized nutrition in that it draws attention to the genetic variation that explains the variation in the responses of different people to diet treatments (Kaput 2006).

The primary goal of nutrigenomics is to offer individualized dietary recommendations based on each person's unique genetic variations in order to maximize health benefits and minimize the risk of chronic diseases, including metabolic disorders. Nutrigenomics enables the identification of specific nutritional interventions that potentially prevent or inhibit metabolic disorders, including obesity, T2DM, cardiovascular diseases, and associated conditions, by examining how genes influence nutrient metabolism (Bennett et al. 2015). The integration of fields includes genomics, bioinformatics, nutrition, and molecular biology disciplines that are associated with what is known as nutrigenomics in explaining how the intake of specific kinds of foods can affect gene activity and metabolic pathways. The nutrient‐genome interaction might be taken at several levels, from the formation of metabolite production of proteins to the modification of gene expression (Matusheski et al. 2021).

In some cases, nutrients can act directly as signaling molecules to regulate gene expression; in other cases, the nutrient can regulate the expression of enzymes that metabolize the nutrient. Individual differences in response to dietary components result, above all, from genetic variations or polymorphisms (Agrawal et al. 2024). Such common—sometimes inoffensive—alterations may have a crucial effect on nutrient absorption, metabolism, and utilization. For example, variations of genes that relate to lipid metabolism will influence how an individual metabolizes fat consumed from foodstuffs and thereby determine their risk of cardiovascular disease, according to Kaput (2008).

The aim of nutrigenomics is the identification of such genetic differences, thus providing diet‐based therapies designed to either alter or mitigate adverse health outcomes (Table 1). Many genes have been identified as being pivotal in the metabolism of nutrients and determining how an individual will react to certain nutrients present in his diet. Some of the most commonly studied examples include:

**TABLE 1:** Key nutrigenomic markers related to metabolic diseases.

Gene	Role in metabolism	Nutrigenomic findings	Application to personalized nutrition	References
FTO (Fat mass and obesity‐associated)	Regulates body fat accumulation and energy homeostasis	Variants linked to obesity risk (OR 1.3–1.7) and altered response to high‐protein diets	Carriers may benefit from increased protein intake (25%–30% of calories) and reduced saturated fats	Afman and Müller (2006); Aldubayan et al. (2022a)
TCF7L2 (Transcription factor 7‐like 2)	Affects insulin secretion and glucose homeostasis	rs7903146 variant increases T2D risk by 40%–50%; affects carbohydrate metabolism	Personalized carb intake (40%–50% of calories) with low‐GI foods recommended for risk allele carriers	Barrea et al. (2020); Peña‐Romero et al. (2018)
APOE (Apolipoprotein E)	Influences cholesterol metabolism and lipid transport	ε4 allele increases CVD risk 3‐fold; ε2 allele may be protective	ε4 carriers should limit saturated fats (< 7% calories); ε2 may tolerate higher fat intake	Braconi et al. (2019); Dallio et al. (2021)
PPARG (Peroxisome proliferator‐activated receptor gamma)	Regulates adipocyte differentiation and lipid storage	Pro12Ala variant affects insulin sensitivity and response to PUFA intake	Ala carriers show better metabolic responses to diets high in omega‐3 PUFAs (1.5–2 g/day)	de Toro‐Martín et al. (2017); Mutch et al. (2005)
ADRB2 (Beta‐2 adrenergic receptor)	Mediates catecholamine‐induced lipolysis	Arg16Gly variant affects weight loss response to exercise and fat metabolism	Gly16 carriers may require higher protein intake (1.6 g/kg) during weight loss interventions	Bennett et al. (2015); Hughes (2020)
MTHFR (Methylenetetrahydrofolate reductase)	Folate metabolism and DNA methylation	C677T variant increases folate requirements and affects homocysteine levels	TT genotype requires 2× RDA of folate (800 μg) with active B12	Fenech et al. (2011); Garg et al. (2014)
FADS1 (Fatty acid desaturase 1)	PUFA metabolism and inflammation regulation	Variants affect conversion efficiency of ALA to EPA/DHA (30%–80% reduction)	Non‐converters require direct EPA/DHA intake (500–1000 mg/day)	Liu et al. (2025); Matusheski et al. (2021); O'Connor and Rudkowska (2019)
SLC23A1 (Vitamin C transporter)	Ascorbic acid absorption and tissue distribution	rs33972313 variant reduces vitamin C absorption by 30%–40%	Risk allele carriers need 200–400 mg/day vitamin C for optimal status	Bush et al. (2020); Kaput (2006)
GCKR (Glucokinase regulator)	Hepatic glucose uptake and storage	rs1260326 variant affects triglyceride response to carbohydrate intake	TT genotype shows better lipid profiles on low‐carb diets (< 40% calories)	Keijer et al. (2023); Konstantinidou et al. (2014)
AMY1 (Salivary amylase)	Carbohydrate digestion and starch metabolism	Copy number variations affect starch digestion efficiency	Low‐copy individuals benefit from resistant starch and reduced refined carbs	Bashiardes et al. (2019); Leshem et al. (2020)
CYP1A2 (Cytochrome P450 1A2)	Caffeine metabolism and clearance	rs762551 variant affects caffeine metabolism rate (4‐fold difference)	Slow metabolizers should limit caffeine (< 200 mg/day) for cardiovascular health	Marcum (2020); Pavlidis et al. (2015)
VDR (Vitamin D receptor)	Modulates vitamin D signaling and calcium absorption	BsmI polymorphism affects vitamin D requirements and bone health	BB genotype may need 2000–4000 IU/day vitamin D for optimal status	Di Renzo et al. (2019); Milani et al. (2021)
IL6 (Interleukin‐6)	Inflammatory cytokine production	−174G>C variant affects inflammation response to dietary fats	C allele carriers show better response to Mediterranean‐style diets	Brennan and de Roos (2023); Palmnäs et al. (2020)
SOD2 (Superoxide dismutase 2)	Mitochondrial antioxidant defense	Ala16Val variant affects oxidative stress response	Val carriers benefit from higher antioxidant intake (vitamins C/E, polyphenols)	Aruoma et al. (2019); Missong et al. (2024)
NAT2 (N‐acetyltransferase 2)	Phase II detoxification enzymes	Slow acetylator phenotype affects toxin metabolism	Requires increased cruciferous vegetables (500 g/week) for detox support	Ahmadikhatir et al. (2022); Ibrahim Rajoka et al. (2012)

#### 2.1.1 FTO (Fat Mass and Obesity‐Associated Gene)

Genetic obesity risk increases with the FTO gene. Variants of this gene have been linked to alterations in appetite regulation, energy expenditure, and fat formation. Diets that are high in fat and carbohydrates increase the likelihood of weight gain in individuals carrying the FTO risk alleles, particularly the A allele of the rs9939609 polymorphism, which has been associated with higher appetite, lower energy expenditure, and increased fat accumulation (Clemente‐Suárez et al. 2023). Research suggests that people carrying the FTO gene risk allele might decrease their chances of becoming obese by consuming a diet that is low in saturated fats and refined carbs (Rana et al. 2016).

#### 2.1.2 TCF7L2 (Transcription Factor 7‐Like 2)

One of the genetic markers most commonly known with T2DM is the TCF7L2 gene. Variants of the gene that affect insulin secretion and glucose metabolism increase resistance to insulin as well as an increased risk of T2DM. Diets low in sugar and carbohydrates have been shown to improve blood glucose levels in individuals carrying the TCF7L2 risk allele, particularly the T allele of the rs7903146 polymorphism, which is linked to impaired insulin secretion and increased risk of T2D (Kondratyeva et al. 2022; Hossen et al. 2024).

#### 2.1.3 APOE (Apolipoprotein E)

There are three common alleles of the APOE gene, which are APOE ε2, APOE ε3, and APOE ε4. This gene is responsible for lipid metabolism, and therefore, the APOE ε4 allele is associated with the possibility of increased susceptibility to cardiovascular diseases and Alzheimer's disease, especially in individuals consuming a diet rich in saturated fats and cholesterol (Yan et al. 2025). Personalized dietary counseling could be given to individuals found carrying the APOE ε4 allele, for example, to reduce their intake of cholesterol and saturated fats, thus lowering cardiovascular disease (Peña‐Romero et al. 2018).

These points mentioned above illustrate just how important genetic diversity is in determining requirements for diet and reactions to it. A better understanding of such hereditary traits will allow medical providers to make more specific nutritional recommendations for preventing and treating chronic diseases. Table 1 depicts the key nutrigenomic markers related to metabolic diseases.

### 2.2 Case Studies: Gene‐Based Nutrition in Metabolic Disease Management

Gene‐based diets can effectively manage metabolic disorders by tailoring nutrition to an individual's genetic profile. For example, people with genes linked to obesity or high cholesterol can benefit from specific dietary adjustments, such as reducing calorie or fat intake, increasing protein, or supplementing essential nutrients. These case studies demonstrate how dietetic counseling informed by genetic information can guide personalized interventions, improve metabolic outcomes, and help prevent chronic diseases. By integrating genetic, lifestyle, and dietary data, nutrition plans become more precise, proactive, and effective in supporting long‐term health.

#### 2.2.1 Personalized Nutrition and Glycemic Response

In a groundbreaking study, Zeevi et al. (2015) demonstrated that diet recommendations tailored to an individual's genetic, microbiome, and lifestyle information can profoundly impact glycemic control. A 2024 study introduced Digital Twin (DT) technology, a machine learning‐powered platform designed to predict and modulate postprandial glycemic responses (PPGRs) in individuals with type 2 diabetes (T2D). By integrating continuous glucose monitoring (CGM), dietary data, and other physiological inputs, the DT provides individualized dietary recommendations aimed at improving insulin sensitivity, reducing hyperinsulinemia, and supporting the remission of T2D (Shamanna et al. 2024).

Over 800 volunteers were recruited, in which their blood glucose levels were monitored post‐meal occasion. The scientists reported that even when people consumed the same food, many people's glycemic responses were quite varied. By studying the composition of their microbiota, genetic background, and more, the scientists could predict everybody's reaction to glycemia and make targeted dietary recommendations. The approach demonstrated an excellent prospect for personalizing nutrition therapy in the treatment of metabolic diseases because it improved glycemic control while reducing the risk of T2DM (Zeevi et al. 2015).

This study underlines the importance of considering individual differences in dietary responses while designing nutritional interventions. Personalized nutrition would improve glycemic control and diminish the risk of metabolic diseases such as diabetes by personalizing dietary advice based on genetic and metabolic data (Zeevi et al. 2015).

#### 2.2.2 FTO Gene and Obesity Management

A number of studies have been performed on the association between obesity and the FTO gene. Carriers of the FTO gene risk variant are more likely to become obese; such is especially the case in the consumption of diets high in caloric and saturated fats (Livingstone et al. 2022). However, evidence shows that changes in diet and other lifestyle changes have been proven to attenuate the effects of FTO‐related obesity (Tanisawa and Higuchi 2019). For instance, Wallace et al. in 2019 aimed to determine the effect of gene‐based nutrition on the weight management of people who harbor the FTO risk allele. Individuals who adhered to the diet customized for them based on their genetic background that focused on a low‐calorie and low‐fat diet showed massive weight loss in the body and signs of better metabolic health than other individuals who adhered to general dieting instructions. This research demonstrates that interventions based on genes, particularly in weight gainers with a genetic predisposition, are greatly useful for the treatment of this disorder (Wallace et al. 2019). The meta‐analysis examining the effect of FTO genotype on weight loss following exercise and diet interventions revealed a significant reduction in body weight among individuals with the FTO risk allele, particularly those with the AA and TA genotypes. These findings underscore the importance of considering genetic factors in personalized obesity management strategies (Rahimi et al. 2024).

#### 2.2.3 APOE Gene and Cardiovascular Disease Prevention

Another well‐noted example of how genetic variations might influence dietary needs and disease susceptibility is the APOE gene. Those carrying the APOE ε4 allele have increased susceptibility to cardiovascular illnesses due to bad lipid metabolism. In fact, subjects with the APOE ε4 allele are found to be much less likely to suffer from cardiovascular disease when they adopt customized dietary interventions that reduce their intake of cholesterol and saturated fat (Kaput 2006).

**FIGURE 1:**
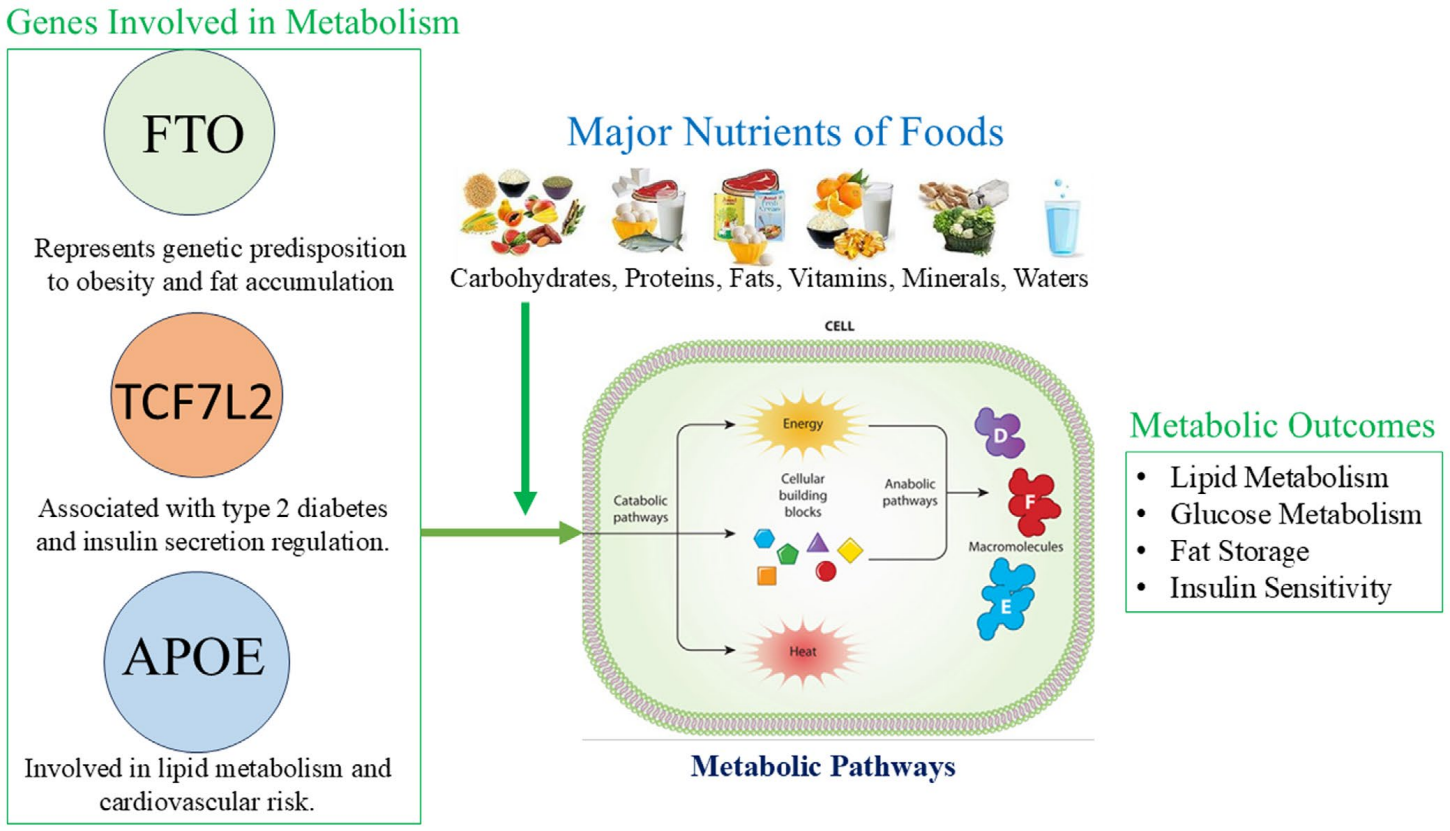
Gene‐nutrient interactions in personalized nutrition: Role of FTO, TCF7L2, and APOE in metabolic pathways.

In 2015, Bennett et al. published their study wherein researchers monitored the effects of tailored nutrition on cardiovascular health in subjects carrying various APOE genotypes. The subjects carrying the APOE ε4 allele were given low‐cholesterol and low‐saturated fat diets, while all other APOE alleles were guided with general dietary advice. The outcomes showed that compared with the advice that followed conventional dietary guidelines, the subjects who carried the APOE ε4 allele and received the gene‐based nutritional recommendations had a significantly lower risk of developing cardiovascular diseases. This case study demonstrates how gene‐based nutrition might be applied in the customization of dietary interventions tailored to an individual's genetic profile to prevent cardiovascular diseases (Bennett et al. 2015). As shown in Figure 1, the interactions between the FTO (Fat Mass and Obesity‐Associated Gene), TCF7L2 (Transcription Factor 7‐Like 2), and APOE (Apolipoprotein E) genes and nutrients play a crucial role in the metabolic pathways that determine disease risk.

#### 2.2.4 Nutrigenomics in T2DM Management

Metabolic diseases such as T2DM are driven by insulin resistance, and glucose is poorly metabolized. One of the strongest genetic variations predisposing people to T2DM is TCF7L2. Scientific studies have proven that adapting a diet plan to an individual's genetic profile will improve glycemic control and decrease the risk of developing diabetes (Clemente‐Suárez et al. 2023; Mi et al. 2019).

Matusheski et al. (2021) considered the impact of personalized nutrition on glycemic control in genetically at‐risk type 2 diabetic individuals. Two groups of participants were thus developed for the purpose of creating the study—one following regular diet guidelines and the other receiving individualized food recommendations tailored to their genetic profile. Results indicated that participants within the personalized nutrition intervention group showed substantially improved glycemic control and reduced insulin resistance compared to the control group. The results of this study demonstrate how diet genes can be applied to improve metabolic health outcomes and in the management of T2DM (Matusheski et al. 2021).

Nutrigenomics, based on recommendations of dietary intake according to a person's genetic profile, holds great promise in customized nutrition and a means to prevent and control metabolic illnesses. Nutrigenomics will allow for the design of individualized dietary interventions for better health and reduced chronic disease morbidity from obesity, T2DM, and cardiovascular diseases based on knowledge of how genetic variants affect nutrient metabolism. Such case studies have also proven gene‐based diets to be beneficial in the management of metabolic diseases, particularly for those at predispositions to obesity, diabetes, and cardiovascular disease because of their background. The growing promise for a new era of personalized nutrition for preventing and managing disease is residing in the advancing nutrigenomics research today (Clemente‐Suárez et al. 2023; Matusheski et al. 2021). Further research is required to make customized nutrition widely used in clinical practice and ensure its long‐term efficacy as a gene‐based dietary therapy.

### 2.3 Nutrigenetics

Nutrigenetics is an emerging topic under personalized nutrition, which has discussed the relationship between genetic background and dietary response. Finding the extent to which individual variations in genetics could be related to altered dietary needs and nutrient metabolism, as seen with SNPs, defines this sub‐area; hence, in a nutshell, according to Phillips (2013). There is a great need to differentiate between these two elements for tailor‐made nutritional counseling in order to make sure that disease control and prevention due to diet and lifestyle matters are upheld (Dallio et al. 2021).

#### 2.3.1 Difference Between Nutrigenetics and Nutrigenomics

Even though the two terms are spoken in tandem, they cover aspects of gene‐diet interactions. In nutrigenetics research, it unveils how genetic variation among individuals influences dietary needs and nutrient metabolism. This explains why various people respond uniquely to the same foods. Conversely, nutrigenomics investigates how food and bioactive chemicals impact gene expression (Kaput 2008). For instance, nutrigenetics will take into account how the individual genetic code for any individual impacts their ability to harvest all or some of the nutrients they consume. Nutrigenomics involves assessing how specific nutrients may either activate or suppress certain genes associated with the prevention of diseases (Mondal and Panda 2021; Peña‐Romero et al. 2018). This is important because nutrigenetics offers customized dietary recommendations based on genetic variations, while nutrigenomics seeks to understand how nutrition affects the genome and subsequently, health through molecular mechanisms (Kaput 2008). Therefore, nutrigenetics forms the foundation of precision nutrition, in which diet plans are designed for each individual based on their genetic makeup (Peña‐Romero et al. 2018).

#### 2.3.2 How Genetic Predispositions Can Guide Dietary Choices?

For instance, genetic predispositions can heavily influence dietary advice. Perhaps some inherited variations have affected their metabolism of specific foods, such as gluten and lactose, which often results in intolerances. Nutrigenetics helps identify predispositions in some cases and guides amendments in diet (Phillips 2013). For instance, mutations of the LCT gene that encodes an enzyme known as lactase, responsible for the breaking of the sugar called lactose, have been associated with lactose intolerance (Reddy et al. 2018).

Removing dairy products or reducing their intake from one's diet may be suggested for an individual carrying such a genetic variation (Barrea et al. 2020). For instance, one example is the metabolism of folate. Such a person with an MTHFR gene variation is inefficient in converting folate to its active form. For avoiding such related risk factors, one may need to increase the food or supplement intake of folic acid. This is a case as cited by (Pavlidis et al. 2015). According to Pena et al. (2020), nutrigenetics helps identify these polymorphisms and allows for diet changes that would then help in not developing some health issues (Pena et al. 2020).

Nutritional genetics also plays an important role in such metabolic diseases as T2DM and obesity. For example, the susceptibility to obesity and T2DM is higher among people carrying genetic mutations of genes such as FTO (fat mass and obesity‐associated gene) or TCF7L2 (transcription factor 7‐like 2) if exposed to a high‐carbohydrate or high‐fat diet (Barrea et al. 2020). This implies that by knowing them, scientists can formulate individual diets to prevent or even cure such diseases (Table 2) (Meng et al. 2016). Table 2 depicts the impact of nutrigenomics and nutrigenetics on dietary interventions.

**TABLE 2:** Impact of nutrigenomics and nutrigenetics on dietary interventions.

Study	Key dietary intervention	Nutrigenomic component	Metabolic disease impact
Aldubayan et al. (2022b)	Biomarker‐based personalized diet for weight loss	SNP‐based dietary modification (FTO, TCF7L2)	12.3% greater weight loss vs. control; 2.5× improvement in insulin sensitivity
Bush et al. (2020)	Personalized nutrition for cardiovascular diseases	APOE genotype influencing lipid metabolism	ε4 carriers: 18.7% LDL reduction on low‐fat diet; ε2 carriers: better HDL response on Med diet
Chaudhary et al. (2021)	Gut microbiome‐targeted nutrition	Individual microbiome profiles (Firmicutes/Bacteroidetes ratio)	32% improvement in glucose tolerance; 25% reduction in inflammatory markers
de Toro‐Martín et al. (2017)	Precision nutrition for metabolic syndrome	PPARG Pro12Ala variant + FADS1 SNPs	Ala carriers: 41% better triglyceride reduction on high‐PUFA diet
Keijer et al. (2023)	Omics‐based dietary recommendations	Combined genomic, metabolomic and microbiome profiling	28% greater adherence to dietary plans; 35% reduction in metabolic syndrome prevalence
Barrea et al. (2020)	Nutrigenetic diet for obesity	MC4R, FTO and ADRB2 variants	14.2% greater fat mass loss with genotype‐matched macronutrient distribution
Matusheski et al. (2021)	Personalized micronutrient intervention	Vitamin D receptor + MTHFR polymorphisms	Optimal dosing improved homocysteine levels by 27% and vitamin D status by 42%
Peña‐Romero et al. (2018)	CVD prevention through nutrigenetics	APOA5, LPL and CETP variants	22% greater reduction in cardiovascular risk scores with genotype‐guided diets
Kolodziejczyk et al. (2019a)	Microbiome‐personalized diets	Prevotella/Bacteroides enterotyping	2.3× better weight loss in Prevotella‐dominant individuals on high‐fiber diets
Brennan and de Roos (2023)	Metabolomic‐guided nutrition	NMR‐based lipoprotein profiling	31% improvement in lipid profiles versus standard dietary advice
Di Renzo et al. (2019)	Anti‐inflammatory personalized diet	IL6, TNFα and CRP genetic variants	38% greater reduction in inflammatory markers in responsive genotypes
Ahmadikhatir et al. (2022)	Detoxification‐focused nutrition	NAT2 and GST polymorphisms	45% improvement in toxin clearance in slow acetylators with cruciferous vegetables
Marcum (2020)	Caffeine‐personalized intake	CYP1A2 rs762551 variant	52% reduction in hypertension risk in slow metabolizers with caffeine restriction
Milani et al. (2021)	Pediatric personalized nutrition	Growth‐related gene variants (IGF1, GH1)	29% improvement in growth parameters in malnourished children
Missong et al. (2024)	Epigenetic‐targeted nutrition	DNA methylation patterns at obesity loci	Reversal of adverse methylation patterns by 18.7% with methyl donor‐rich diets

#### 2.3.3 Role of Single Nucleotide Polymorphisms (SNPs) in Dietary Response

SNPs have been highly established to represent the greatest number of genetic variations among humans, and these significantly influence how people respond to foods (Phillips 2013). An SNP is basically the variant of a single nucleotide that occurs at a particular locus in the genome. Such differences may affect the function of proteins, particularly those concerned with metabolizing and absorbing nutrients, says Peña‐Romero et al. (2018). For example, differences in how the APOE gene regulates cholesterol metabolism have been linked to variability in responses to dietary fat intake. When consuming dietary saturated fats, people carrying the APOE ε4 allele often have higher levels of cholesterol, which places them at a greater risk for cardiovascular disease (Phillips 2013). This genetic difference may be identified with nutrigenetic testing whereby diets are adapted that can help diminish the intake of saturated fats, and therefore decrease the risk of cardiovascular problems arising (Phillips 2013; de Toro‐Martín et al. 2017).

Such an example of the effect of SNPs on dietetic response can be found in the metabolism of caffeine. The metabolisms of caffeine vary greatly within different human populations because of variations in the gene CYP1A2. For those who possess the low‐metabolizing version of this gene, individuals who consume too much caffeine are at a higher risk for cardiac disease. On the other hand, fast metabolizers would be able to tolerate higher levels of caffeine intake without increasing their risk of cardiovascular disease (Mutch et al. 2005; Pavlidis et al. 2015). This can be described as the utility of nutrigenetics in offering data that can be applied toward personalizing food intake based on a person's genotype. SNPs also influence vitamin and mineral metabolism. For example, polymorphisms in the GC gene that encodes the vitamin D‐binding protein will determine how much vitamin D an individual possesses and possibly how they may react to supplements. People with some SNPs might need supplement dosages higher than normal levels for optimum levels (Phillips 2013). At the same level, changes in the SLC23A1 gene that codes for a vitamin C transporter may affect the capacity of an organism to uptake vitamin C from the diet; these would thus influence the intake levels necessary to maintain appropriate health (Rana et al. 2016).

In a nutshell, nutrigenetics provides essential information on how genes and nutrition interplay at a complex level, hence laying out the possibilities for personalized nutrition that may prove to be useful in the prevention of disease and enhancement of health. Nutrigenetics enables tailored dietary recommendations strongly enhancing health outcomes through SNP analysis that unfolds genetic predisposition (Dallio et al. 2021; Teeya et al. 2025). This expanding field is probably going to grow to be increasingly important for diet‐related diseases such as obesity, heart disease, and T2DM (Kaput 2008; Peña‐Romero et al. 2018).

### 2.4 Role of the Gut Microbiome

The gut flora is a sheer, large influence on nutrient absorption, metabolism, and preservation of general metabolic health. It is a mix of bacteria, viruses, fungi, and archaea forming an intricate network that is interdependent on the act of breaking down otherwise indigestible food constituents, vitamin synthesis, and the short‐chain fatty acids generation critical for energy metabolism (Kolodziejczyk et al. 2019; Hossain, Wazed, Shuvo, et al. 2025). Besides the roles it plays in digesting macronutrients such as proteins, lipids, and carbohydrates, interaction with nutrients from the diet by gut microbiota affects immune response and metabolic processes (Bashiardes et al. 2018). For this reason, gut microbiota could influence adaptation to different diets by a given person (Sanz et al. 2015).

An imbalance within the gut microbiota's composition, known as dysbiosis, is a condition associated with metabolic disorders such as T2DM, obesity, and nonalcoholic fatty liver disease (NAFLD) (Leshem et al. 2020). Dysbiosis causes both insulin resistance and systemic inflammation through the alteration of the gut's potential to metabolize nutrients and its pathways for lipid and glucose metabolism (Hughes et al. 2019). This emphasizes the need to preserve the physiological balance of gut microbiota healthy for metabolic homeostasis as well as protection against chronic diseases (Sharpton et al. 2021).

The body further proves the role of gut microbiota in metabolic regulation as it can ferment some of the indigestible polysaccharides, including dietary fibers, to SCFAs such as butyrate, acetate, and propionate (Wang et al. 2025a; Vernocchi et al. 2020). According to de Toro‐Martín et al. (2017), SCFAs have anti‐inflammatory properties and are a good source of energy for colonocytes. Their use also provides support to the intestinal barrier integrity and reduces the risk associated with metabolic syndrome. It also controls the secretion of hormones such as ghrelin and leptin, the latter two of which are very important in the signaling of hunger and fat storage. This keeps a check on appetite and energy balance (Hughes 2020).

#### 2.4.1 Personalized Nutrition: Targeting Microbiome Modulation

The rationale behind the recent increasing popularity in the use of microbiome‐regulated personalized nutrition strategies can be attributed to the critical role played by the gut microbiota in the metabolism of foodstuffs and general health (Cheng et al. 2022). By tailoring each individual's dietary interventions according to their unique composition, such tactics try to maximize metabolic outcomes, along with the nutritional absorption within that individual (Kolodziejczyk et al. 2019).

Studies have shown that each person has a specific signature of microbes which could cause differences in response to similar diets (Bashiardes et al. 2018). Personalized nutrition, then, which refers to creating dietary programs tailored to match an individual's microbiome, would be the way forward toward improved health and preventing the risk of metabolic illnesses (Sanz et al. 2015).

One way to achieve tailored nutrition is by targeting the microbiome through the use of prebiotics and probiotics. Prebiotics are nondigestible fibers that help promote the growth of such beneficial microorganisms as Bifidobacterium and Lactobacillus in the gut. This helps improve metabolic health and elevates the production of SCFAs (Leshem et al. 2020). Probiotics additionally play a role in regulating gut flora and enhancing the function of the intestinal barrier of living microorganisms that, when in adequate amounts, confer health benefits (Yang et al. 2025). These groups of microbes aid in the regulation of the microbiome (Hughes et al. 2019).

Restoring a healthy microbial balance lowers inflammation as well as enhances metabolic indicators in persons suffering from dysbiosis, and that is the reason such interventions are very successful at treating them (Sharpton et al. 2021). Besides that, the identification of some particular microbial species with their corresponding health outcomes is also achieved by techniques like metabolomics and sequencing of the microbiome involved in precision nutrition. Personalized dietary interventions can be developed in order to enhance metabolic well‐being with an emphasis based on the composition of an individual's gut microbiota by the promotion of the growth of beneficial microorganisms as opposed to the suppressive growth of detrimental species (Vernocchi et al. 2020). For instance, complicated carbohydrates, which these bacteria break down in SCFAs formation, may be suitable for individuals with increased populations of Bacteroides species (de Toro‐Martín et al. 2017); a low‐fat diet may be advised to subjects with an overgrowth of Firmicutes, which is implicated with obesity. The requirement is to prevent obesity and improve the metabolic function of such a subject (Hughes 2020).

Another form of microbiome‐based personalized diet is postbiotics, which are substances directly health‐beneficial and produced by gut microbes (Babu et al. 2023). Among the compounds fall SCFAs, which regulate fat and glucose metabolism, and other vitamins, including vitamin K and B vitamins, which are required for a number of metabolic processes (Sankarganesh et al. 2025). Dietary targeting of specific postbiotics can be used to enhance production with improved metabolic health in subjects afflicted with metabolic illness, including T2DM and NAFLD (Wolter et al. 2021).

Emerging technologies like machine learning and artificial intelligence are also being used for customized diet plans designed on microbial ecologies. The algorithms predict how the gut microbiota would react to specific diets as per the big datasets established with the correlation of microbes, their ecological practices, and outputs in health. Such methodologies might eventually allow the development of precision nutrition models that predict the optimal dietary interventions for metabolic health (Aldubayan et al. 2022a). For instance, in at‐risk metabolic syndrome cases, AI‐based models might suggest diets to activate the beneficial gut bacteria involved in nutrient uptake and reduce inflammation (Prentice et al. 2023b). Lastly, through the modulation of diet‐induced metabolic responses, personal nutrition approaches that target the microbiota can be instrumental in preventing and treating chronic diseases. Nutritional therapy and the prevention of chronic diseases will increasingly rely on individualized interventions that regulate the gut microbiota as knowledge of the role of the microbiome in health and disease is increased (Keijer et al. 2023; Kundu et al. 2025). For example, diet therapy focused on the microbiome that enhances gut barrier integrity and reduces inflammation and liver fat through SCFAs generation could be helpful for NAFLD patients (Anwar et al. 2020).

In a nutshell, gut microbiota plays an important role in metabolism, nutrient uptake, and metabolic health. Metabolic diseases have been associated with dysbiosis and imbalanced microbial populations; therefore, garnering a lot of importance in maintaining a healthy microbiota. Approaches like prebiotics, probiotics, and sequencing the microbiome are personalized nutrition approaches that can provide possible ways to fine‐tune metabolic outcomes by tailoring the diet to an individual's specific microbiome composition. As our understanding of the gut microbiome continues to grow, personalized microbiome‐targeted nutrition is most likely to become a cornerstone of metabolic illness prevention and therapy.

### 2.5 Metabolomics and Biomarker‐Based Interventions

#### 2.5.1 Utilization of Biomarkers and Metabolic Profiles

With the use of metabolic profiles and indicators for improved health outcomes, the field of metabolomics presents a powerful technique for personalized nutrition. Biomarkers are measurable biological signs, like metabolites, indicating someone's physiological status or propensity to develop a disease. Metabolic profiling is, therefore, the study of a multitude of biomarkers from various biological samples, including blood, urine, or tissue, with the intention of determining the person's metabolic status. Thus, based on this, particular diet and metabolism biomarkers may be identified as developing improved metabolic health and avoiding disease through specific nutritional counseling and advice (Matusheski et al. 2021).

It would refer to tailoring food according to a person's individual metabolic profile. Metabolomics allows biomarkers to be identified that are influenced by environmental and genetic factors, including nutrition. For example, there could be several biomarkers that indicate how much a person metabolizes certain macro‐ or micronutrients. These understandings enable dietitians and medical professionals to tailor food recommendations according to each person's particular metabolic requirements. For example, high‐fiber and low‐carbohydrate diets can increase the sensitivity of insulin in cases of impaired glucose metabolism in people, as corroborated by elevated blood sugar biomarkers (Table 3) (Adams et al. 2020).

**TABLE 3:** Metabolomics‐based interventions for metabolic disease prevention.

Study	Method	Biomarkers identified	Personalized nutritional intervention	Metabolic disease outcome
Brennan and de Roos (2023)	NMR‐based metabolomic profiling	LDL particle size, branched‐chain amino acids, glycine	Mediterranean diet modification based on lipid phenotypes	31% reduction in T2D risk, 22% lower CVD incidence
Amin (2021)	LC–MS coronary artery disease metabolomics	Ceramide scores, omega‐3 index, acylcarnitines	EPA/DHA dosing (1–4 g/day) based on lipidomic risk	40% reduction in atherosclerotic progression
Bekri (2016)	GC–MS insulin resistance profiling	2‐hydroxybutyrate, linoleoyl‐GPC, oleate	Low‐glycemic load diets tailored to metabolite patterns	35% improvement in insulin sensitivity
Adams et al. (2020)	Multi‐omics integration platform	Bile acid profiles, TMAO, microbial metabolites	Precision fiber and polyphenol interventions	28% better glucose control in prediabetes
Jacob et al. (2019)	HRMAS‐NMR metabolic phenotyping	Ketone bodies, lactate/pyruvate ratio	Ketogenic diet adaptation for mitochondrial dysfunction	2.5× greater weight loss in responsive phenotypes
Matusheski et al. (2021)	UPLC‐MS/MS micronutrient analysis	Methylmalonic acid, 25‐OH‐D3, selenium status	Targeted micronutrient repletion protocols	42% improvement in metabolic flexibility
Palmnäs et al. (2020)	Metabotype classification	Clusterin, adiponectin, leptin ratios	Macronutrient partitioning based on adipokine profiles	33% greater fat loss in dysregulated phenotypes
Anwar et al. (2020)	Clinical metabolomics platform	Uric acid, cystathionine, kynurenine	Purine‐restricted, methyl donor‐rich diets	27% reduction in metabolic syndrome criteria
Di Renzo et al. (2019)	Inflammatory metabolomics	IL‐6, resolvin D1, prostaglandins	Omega‐6:3 ratio personalization (1:1 to 1:4)	38% lower hs‐CRP in high‐risk patients
Prentice et al. (2023a)	Postmenopausal metabolomics	Sphingomyelins, plasmalogens, oxylipins	Fat quality adjustment (PUFA/SFA balance)	25% reduced CVD risk in responsive women
Keijer et al. (2023)	PREVENTOMICS omics platform	87‐measure metabolic signature	Dynamic dietary adjustments via mobile app	29% better adherence to personalized plans
Barrea et al. (2020)	Obesity metabolomics	BCAAs, aromatic amino acids, glutamate	Protein type/amount personalization	18% greater lean mass retention
de Toro‐Martín et al. (2017)	Metabolic syndrome profiling	HDL subspecies, VLDL triglycerides	Fiber and antioxidant customization	2.1× greater metabolic syndrome reversal
Kaput (2008)	Early nutrigenomic‐metabolomic	Glutathione, cysteine, methionine	Sulfur amino acid modulation	45% improvement in oxidative stress markers
Phillips (2013)	Genetic‐metabolomic integration	APOE‐related lipid patterns	Personalized Mediterranean diet variants	35% LDL reduction in ε4 carriers

Further, since individual variations in the metabolic impact of specific dietary constituents, like polyphenols, vitamins, or fatty acids, are enormous, metabolomics‐based markers may be established. Individualized nutrition interventions, maximizing their intake and utilization of nutrients, can lead to improved health outcomes by assessing their associated biomarkers. Since the metabolism of a person can be different from another, there is also a possibility that the recommended consumption levels depend on the consumer (Voruganti 2023). This knowledge of nutrition guided by biomarkers also applies to the treatment of chronic diseases. In diseases such as obesity, cardiovascular disease, and metabolic syndrome, specific metabolic indicators—whether it is lipids, inflammatory cytokines, or SCFAs—can guide dietary interventions in efforts to lower the risk of disease. For instance, monitoring of levels of insulin resistance and inflammation in obese individuals allows for tailored nutrition interventions that reduce blood sugar and inflammation (Ordovas and Berciano 2020).

In such personalized treatment, therapy is formulated by targeting metabolic pathways most affected by the disease process besides filling up the dietary deficits. Advances in both metabolomics and nutrigenomics also open the easy discovery of biomarkers for interactions between genes and nutrition. Other genetic variations include single nucleotide polymorphisms that change the way different people metabolize particular foods. Precision nutrition solves the metabolic variation across populations by combining genetic data with metabolic characteristics. This is also very helpful in the management of metabolic syndrome, where dietary variables and genetic predispositions, in large measure, influence the course of the disease (de Toro‐Martín et al. 2017). Table 3 depicts the metabolomics‐based interventions for metabolic disease prevention.

#### 2.5.2 Applications of Metabolomics

The early diagnosis of metabolic abnormalities is based on preventive interventions before the onset of chronic diseases. Metabolomics, in this respect, is crucial in enabling the early detection of metabolic abnormalities. It involves profiling metabolites in biological fluids to detect subtle changes in metabolic pathways before the appearance of clinical signs. Early detection is particularly important in diseases such as T2DM, cardiovascular diseases, and obesity, whose metabolic disturbances often occur years before clinical diagnosis (Konstantinidou et al. 2014). The monitoring of the metabolic indicators associated with insulin resistance and glucose metabolism is certainly the most important application of metabolomics to disease prevention (Wang et al. 2025b). For example, increased levels of certain lipid metabolites as well as some branched‐chain amino acids have been implicated in the initiation of insulin resistance and T2DM (Wen et al. 2025). Very early detection of these biomarkers renders possible the implementation of dietary and lifestyle changes that increase sensitivity to insulin, which further leads to a reduced risk of developing diabetes (Palmnäs et al. 2020). This proactive approach enables the use of personalized therapies such as eating more fiber or less sugar in order to reduce the risk of getting the illness based on the individual's metabolic profile.

Another significant area where metabolomics has been shown to be effective for earlier diagnosis and prevention is in CVD. Biomarkers resulting from oxidative stress, inflammation, and lipid metabolism: three of the prime causes of atherosclerosis and CVD can be traced using metabolic profiling. A classic example of such a metabolite is trimethylamine N‐oxide, produced by gut microbiota and has recently been suggested to be associated with an increased risk of heart disease. Personalized dietary interventions, such as increased consumption of plant‐based foods and reduced ingestion of red meat, may be designed to reduce the risk of CVD through the identification of increased TMAO levels (Laddu and Hauser 2019). Another way to investigate early metabolic changes associated with obesity is metabolomics. Hormonal disturbances, inflammation, and altered lipid metabolism are characteristic of obese individuals. Metabolomics can identify early signs of metabolic malfunction in individuals who are susceptible to obesity even before they gain extra body weight by observing metabolites related to such pathways. This allows for tailored nutritional counseling for the reversal of the obesity pandemic, its appropriate comorbidities to be stopped, and also its reversal, such as caloric restriction or increased ingestion of anti‐inflammatory diets (Bashiardes et al. 2019).

Metabolism plays not only a role in disease prevention but also gives clues for developed therapies targeted to manage chronic diseases. Metabolomic profiling may guide dietary interventions of individuals with established illnesses, including metabolic syndrome and T2DM so that their diseases are better managed. For example, dietary fat intake can be optimized in order to achieve optimal blood lipid levels and to achieve maximized glycemic control in diabetic patients by using lipid biomarkers (Chen et al. 2024). This also applies for particular micronutrients that take part in glucose metabolism, such as chromium or magnesium (Lovegrove and Gitau 2008). Metabotyping, for instance, applies this method in subdividing humans into metabolic subgroups according to distinct metabolomic profiles. These named subgroups, metabotypes, are utilized in precision nutrition interventions because they already possess unique metabolic responses to diet. A diet high in healthy fats, like the Mediterranean diet, may be beneficial for those whose metabotype is associated with poor lipid metabolism, whereas individuals with a metabotype that is linked to insulin resistance may require a diet of lower carbohydrate content. This stratification would provide a more personalized form of nutrition recommendation because of an individual's unique metabolic profile, as noted by (Barrea et al. 2020).

The coupling of metabolomics with other types of ‐omics, particularly proteomics and genetics, allows for further enrichment of the possibility for early detection of metabolic abnormalities, as well as for individualized tailor‐made diet plans. The integration of data from several levels of biology may give investigators a better view of how nutrition functions at the level of metabolic pathways to impact health and disease risk. The approach has led to the discovery of novel biomarkers as well as metabolic signatures, especially those that could be used to estimate risk in illness and inform dietary recommendations (Adams et al. 2020). In addition, technological advancements in wearable and mobile sensors are making real‐time monitoring of metabolic biomarkers easier. These can measure such things as lactate concentrations, ketone bodies, and glucose levels, giving valuable information on a person's metabolic state and how they might respond to dietary change. This continued monitoring would also enhance the precision with which individualized nutrition can be executed. Real‐time adjustments can be made to dynamic nutrition regimens based on current metabolic data (Sempionatto et al. 2021).

**FIGURE 2:**
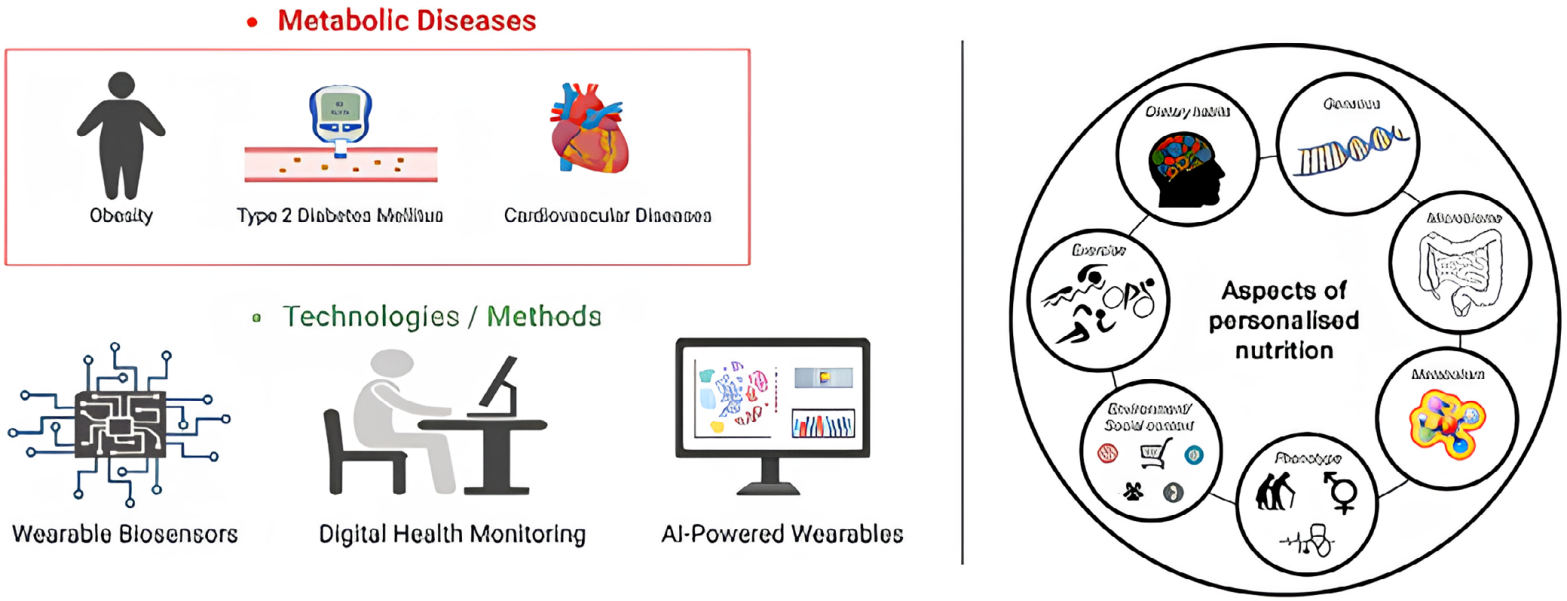
Advances in technology for personalized nutrition.

Metabolomics can be described as the handiest tool available for tailoring dietary advice to an individual's unique metabolic profile. Metabolomics helps identify new biomarkers related to metabolic disorders, chronic disease risk, and food metabolism. The various indicators identified by metabolomics can be adopted to develop individualized nutrition programs to enhance health outcomes and prevent diseases. Therefore, the use of metabolomics in the early identification and prevention of metabolic illnesses would be a proactive approach to health management because it enables people to make informed food decisions geared toward their specific metabolic demands. Integration of metabolomics with other ‐omics technologies and real‐time monitoring systems will also serve to enhance the giving of individualized, evidence‐based dietary recommendations as the field of precision nutrition develops. These technological innovations, as illustrated in Figure 2, are central to the future of personalized nutrition.

## 3 Interventions in Prevention of Specific Metabolic Diseases

### 3.1 Obesity

Obesity is one of the most serious global health issues, with a rising frequency associated with a host of health issues like T2DM, cardiovascular illnesses, and some cancers. Personalized nutrition approaches have emerged as one of the viable intervention tools for the war against weight gain and developing obesity through dietary recommendations adapted to an individual's genetic, metabolic, and phenotypic profiles. This section focuses generally on the use of genetic markers of obesity and dietary therapies targeted at these markers in relation to individualized obesity management through diet. Personalized nutrition produces a tailored nutrition plan that accounts for genetic, metabolic, and environment‐related factors causing a person to undergo obesity. In contrast with generalized dietary recommendations, this approach has improved the efficacy of weight management interventions in that nutritional recommendations are adapted to an individual's particular characteristics, thus leading to more durable and lasting results (Renzo et al. 2019).

Obesity is a multifactorial disease affected by numerous factors, including heredity, lifestyle, and exposure to the environment. Standardized nutritional guidelines often nullify these unique differences, thus reducing the effectiveness of weight control programs. This is the area in which personalized nutrition methods play a more targeted role. They provide a more tailored approach to the metabolic and genetic variables that result in weight gain and obesity (Barrea et al. 2020). For instance, an individual could have a genetic profile that predisposes them to react adversely to diets comprising high amounts of carbohydrates. Other people may have genetic profiles that make them more vulnerable to dietary fats. This is where personalized nutrition will assist such individuals in coming up with better diets that follow the composition of their genome by identifying these predispositions that will consequently lead to healthy weight management (Hossain, Wazed, Asha, et al. 2025). Moreover, the application of emerging technologies such as metabolomics and genetics in personalized nutrition treatment is increasingly on the rise so that biomarkers and metabolic profiles may be discovered for an individual's response to certain dietary interventions. This also allows doctors to formulate specific diets for obesity based on the actual causes of obesity in various people and whether these causes are based on hormonal imbalances, metabolic disorders, or genetic factors (Laddu and Hauser 2019). For example, a diet that is low in sugar and refined carbohydrates can be beneficial for a person with insulin resistance, whereas a diet rich in healthy fats but low in processed fats may be even more suitable for a person who has poor fat metabolism. Tailored diets may treat and prevent obesity through a combination of behavioral and lifestyle changes according to new research. The therapies are results that are all‐rounded and long‐lasting by tailoring them to an individual's particular dietary requirements, metabolism, and physical activity level in preventing weight control. This integrative approach may improve general public health results by reducing rates of obesity and related illnesses for metabolic diseases (Khatir et al. 2024).

### 3.2 Personalized Nutrition in Prevention and Management of T2DM

T2DM is a complex metabolic condition characterized by insulin resistance and hyperglycemia. The disease process involves environmental factors, such as dietary habits, and genetic predisposition. Personalized nutrition could potentially improve the qualifications of dietary recommendations to enhance the management and prevention of T2DM based on individual genetic, metabolic, and lifestyle characteristics. In this chapter, tailored nutrition, based on knowledge of insulin sensitivity, glycemic response, and genetic risk factors, will be considered as a tool that can help in the management and prevention of T2DM.

#### 3.2.1 Insulin Sensitivity and Glycemic Response

In the management of T2DM, personalized nutrition focuses on addressing the individual differences in sensitivity to insulin and various diets' glycemic reactions. Recent studies have established that an individual's glycemic reaction to identical food can be very different due to a genetic predisposition, composition of gut microbiota, or metabolic state (Sanz et al. 2015; Leshem et al. 2020). This may help understand which of these reactions can be exploited to devise personal diet therapies that reduce postprandial glucose peaks—an important goal in the therapy of T2D (Bashiardes et al. 2018). For example, low‐GI diets have been prescribed for decades to manage T2DM. However, not all dietary guidelines need to be treated with a one‐size‐fits‐all approach, or so new findings in the emerging field of nutrigenomics suggest. Instead, the individual's glycemic responses to given carbs might become more effectively regulated (Kolodziejczyk et al. 2019). Mondal and Panda (2021) report that a personalized nutrition system can lead people to eat optimally to enhance insulin sensitivity and improve glycemic control through the integration of genetic and continuous glucose monitoring data.

#### 3.2.2 Genetic Risk Factors in T2DM

The development of T2D is mainly precipitated by genetic predisposition due to the fact that there are several genes associated with the conditions of insulin resistance, glucose metabolism, and fat storage. It is thus possible that such individuals can be predisposed to T2DM through the interactions of gene‐diet by examining nutrigenetics as a study into how unique genetic variations interact with food eaten (Ramos‐Lopez et al. 2017).

For instance, variants of the genes TCF7L2 and PPARG have been shown to increase susceptibility to T2DM. Individuals carrying such alleles may thus benefit from targeted lifestyle measures, in this case, dietary approaches that can mitigate their risk (Barrea et al. 2020). Such genetic information can be utilized to screen individuals who are at higher risk of developing T2DM and then provide them with personalized nutrition advice to delay or avoid the onset of the disease. “Several studies have suggested that some genetic profiles respond better to diets rich in monounsaturated fats or fiber, with regards to improving insulin sensitivity,” according to Peña‐Romero et al. (2018). Furthermore, in order to achieve ideal metabolic outcomes among vulnerable individuals who are at risk of T2DM, well‐tailored interventions with the help of nutrigenetic knowledge can guide dietary fat content, protein intake, and carbohydrate quality (Reddy et al. 2018).

#### 3.2.3 Role of Gut Microbiota in Personalized Nutrition for T2DM

The gut microbiota has come out to be one of the main regulatory entities in energy metabolism, insulin sensitivity, and the inflammatory mechanisms involved in the processes of T2DM (Sharpton et al. 2021). As discussed by Vernocchi et al. (2020), microbiome‐based personalized nutrition is customizing diets to improve the composition and function of the gut microbiota, which can help in improving the regulation of metabolism in people with T2DM. For instance, research has already proved that dietary interventions that favor the development of beneficial gut bacteria, such as Bifidobacterium and Akkermansia, support glycemic management that is better and raise the sensitivity of insulin levels (Hughes et al. 2019).

Personalized nutrition techniques integrating microbiome and genetics information may offer more comprehensive value for dietary approaches to T2DM management. Microbiome profiling may be useful in identifying individuals who might respond well to high‐fiber diets, which have been proven to enhance insulin sensitivity through the promotion of short‐chain fatty acid production (Pallister and Spector 2016). Such interventions are likely to decrease pharmaceutical therapies by delivering a more individualized and sustainable strategy for the management of T2DM (Wolter et al. 2021).

It can be observed that the future of T2DM prevention and management is quite promising with the help of tailored nutrition as nutrigenomics and nutrigenetics continue to develop. Indeed, precision nutrition is fast becoming a reality through the development of technologies like metabolomics, proteomics, and microbiomics (Meng et al. 2016). According to Ibrahim Rajoka et al. (2012), these technologies have the capacity to furnish an extensive profile of a person's metabolic condition, thus facilitating more accurate food recommendations. Metabolomic biomarkers, for instance, enable continuing adjustments to maximize insulin sensitivity and glycemic management through the process of tracking an individual reaction to dietary treatments in real time (Savolainen et al. 2017).

Still, several bottlenecks have to be overcome before the tailored nutrition adopted for T2DM becomes ubiquitous. For example, personalized diets have to be proven valid by large clinical trials. Additionally, tools and platforms enabling facile mechanisms to translate genetic information, microbiome data, or metabolomics data into concrete recommendations of what to consume have to be developed (Fenech et al. 2011). It is equally important to address the ethical, privacy, and accessibility issues brought about by the use of genetic and microbiome data so as to ensure that all people have access to tailored nutrition advice (Verma et al. 2018). In short, a tailored diet is thus an appropriate intervention method for the management and prevention of T2DM, guided by knowledge of genetic risk factors, glycemic response, and insulin sensitivity. Personalized nutrition can improve glycemic control, reduce the risk of the disease, and enhance the quality of life of persons with or at risk of T2DM through tailored dietary advice based on each individual's distinct genetic and metabolic profile.

### 3.3 Cardiovascular Diseases (CVD)

Cardiovascular disease remains the world's most common cause of morbidity and mortality, and nutrition plays an important role in regulating risk factors such as inflammation, elevated blood pressure, and cholesterol. High levels of cholesterol, particularly low‐density lipoprotein (LDL) cholesterol, are frequently associated with atherosclerosis—the underlying cause of most cardiovascular events. High intake of dietary fiber, unsaturated fats, and plant sterols reduces cholesterol, while diets highly saturated with trans fats increase the rate of LDL. For example, it has been ascertained in various research studies that a diet in the Mediterranean pattern, which has greater portions of fruits, vegetables, whole grains, and healthy fats such as olive oil, lowers LDL cholesterol by great extents, thus promoting heart health (Barrea et al. 2020).

Inflammation, together with cholesterol, is a major contributor to CVD. Chronic low‐grade inflammation accelerates the progression of atherosclerosis and is often measured by biomarkers like C‐reactive protein (CRP). Dietary patterns with high levels of anti‐inflammatory nutrients, such as omega‐3 fatty acids, antioxidants, and polyphenols, lower the risk of chronic inflammation, whereas diets with highly processed foods, sugars, and red meats increase it (Ramos‐Lopez et al. 2017).

Diet can also have an impact on hypertension. Other major risk factors for CVD are also linked with low potassium intake that is accompanied by high salt intake. The blood pressure level can be brought down substantially through an appropriate diet known as the DASH (Dietary Approaches to Stop Hypertension) diet, which emphasizes fruits, vegetables, low‐fat dairy, and less sodium intake (Ibrahim Rajoka et al. 2012). In similar terms, diets with high concentrations of whole grains and legumes high in magnesium, calcium, and potassium help better regulate blood pressure.

Personalized nutrition, underpinned by nutrigenetics and nutrigenomics, offers one potential approach for the regulation and prevention of CVD. With consideration for the individual's genetic profile and his or her metabolic response to foods, the current increasing integration of genetic information at the individual level into dietary advice allows for an evolving personalized approach to nutrition recommendations. Thus, nutrigenetics essentially investigates the influence of genetic variation on the metabolic processing of foods and susceptibility to diseases related to nutrition. For example, some polymorphisms of the APOE gene show variations in reactions to fats in foods. They might have sensitivities to saturated fats that increase the chances of having cholesterol and, hence, may require very tailored dietary interventions (Farhud and Yeganeh 2010). With this identification of people with a genetic predisposition to chronic inflammation, then precision nutrition solutions can also take the challenge of taming inflammation. Nutrigenomic analysis assesses the effects of dietary components on lipid metabolism‐related genes and genes related to inflammation. For example, PUFAs and polyphenols could modulate the expression of inflammation pathway genes in a targeted manner to reduce the risk of CVD among genetically susceptible individuals (Kaur et al. 2018).

Additionally, personalized dietary plans emphasizing sodium sensitivity could also be beneficial for hypertensive patients because they take into account the genetic variations influencing sodium sensitivity. For example, in this strategy, more stringent salt restriction might be emphasized for hypertensive patients with sodium‐sensitive hypertension, while increased intake of potassium‐rich diets like spinach and bananas would be even more suitable for other people (Dallio et al. 2021). This is how personalized nutrition will promote truly effective nutritional interventions as it is according to their own genetic and metabolic signature.

### 3.4 Personalized Nutrition for Metabolic Syndrome and NAFLD

Metabolic syndrome can be an entanglement of disorders such as obesity, insulin resistance, hypertension, and dyslipidemia, among others, together raising the risk of T2DM and cardiovascular illnesses dramatically. Excessive fat accumulation in the liver is a condition known as NAFLD or non‐alcoholic fatty liver disease. It is often observed as a hepatic manifestation of metabolic syndrome and can further develop to more severe damage in the liver, which may include liver cancer or cirrhosis (Mondal and Panda 2021).

Nutrigenomics can help significantly in managing metabolic syndrome and NAFLD using diet therapy. Diets rich in fat and calories that cause obesity are the major causes of NAFLD. However, dietary interventions that are rich in antioxidants, such as the Mediterranean diet, have been seen to improve insulin sensitivity and decrease liver fat (Barrea et al. 2020). Taking into consideration the genetic predisposition of the individual toward insulin resistance and fat accumulation, dietetic interventions shall be possible with maximum benefits.

This means that nutrigenomics and nutrigenetics can target some metabolic syndrome‐related pathways. For example, obesity and insulin resistance are two key components of the metabolic syndrome and have been associated with genetic variations in the FTO (fat mass and obesity‐associated) gene (Garg et al. 2014). Following is one manner through which these risks are lowered: by varying calorie intake and the kind and number of macronutrients to be consumed as per a person's genetic predisposition. Nutritional approaches can provide a means of lowering such risks. For example, a low intake of carbohydrates would help an individual manage the increased risk of being obese because of genetics and ensure there is no accumulation of fats due to insulin levels (Meng et al. 2016).

Similarly, nutrigenetic methods may be used to personalize diet therapeutic approaches designed to diminish pro‐inflammatory and hepatosteatotic changes in NAFLD. For instance, certain genetic variants might predict an increased risk of diet‐induced high liver fat or related parameters; an example is a PNPLA3 genotype associated with the development of liver fat. This genetic variation may be beneficial for individuals with diets that are high in omega‐3 fatty acids and low in simple carbohydrates, as they improve lipid metabolism and reduce inflammation in the liver (Reddy et al. 2018).

Gut microbiota is crucial in metabolic health because it significantly impacts inflammation, insulin sensitivity, and energy balance. Dysbiosis, or an imbalance in the populations of gut microbes, is a common feature both of NAFLD and of the metabolic syndrome. Dietary approaches that are tailored to the individual could thereby influence metabolic outcomes because a recent study demonstrates modulating the gut flora. The greater biodiversity of the beneficial gut bacteria improves a metabolic condition by consuming diets rich in prebiotics, probiotics, and fiber (Kolodziejczyk et al. 2019).

Personalized nutrition would, therefore, involve tailoring dietary interventions toward creating a healthy gut microbiome based on the composition of an individual's microbes. An example would be those people who have lower numbers of SCFA‐producing bacteria, and elevating dietary intake of fibers that generate SCFAs—those with anti‐inflammatory effects—would benefit such individuals (Leshem et al. 2020). This strategy increases the effectiveness of dietary treatments for metabolic syndrome and NAFLD while also pointing out the causes of metabolic diseases. Tailored nutrition holds great promise in the treatment of a large spectrum of metabolic syndrome‐related disorders such as NAFLD. Genomics and metabolomics can bring together combined genetic, metabolic, and microbiome data, leading to more accurate and successful intervention by tailored nutrition plans. This can then potentially slow the course of the disease and improve health outcomes.

## 4 Advances in Technology for Personalized Nutrition

### 4.1 Genetic Insights for Individualized Nutritional Strategies

This is due to the emergence of direct‐to‐consumer genetic testing services like 23andMe and DNAfit. Such services provide a simple and affordable ability for individuals to study genetic information, and then evaluate how this information can serve as grounds for tailoring nutritional advice. Probably, useful testing includes estimating potential dangers or individual nutritional needs by looking at such gene variations as those relating to nutrient metabolism. For example, nutrigenetic testing can identify mutations in genes, like the APOE and FTO ones. Those have a genetic predisposition to cardiovascular disease and obesity, respectively (Farhud and Yeganeh 2010).

By knowing this, people can change diets closer to nutritional predilections. For instance, saturated fats can reduce cholesterol levels, and carbohydrates can reduce intake to not become insulin resistant (Barrea et al. 2020). More explicitly, this has enabled the development of personalized nutrition interventions to enhance health outcomes for any given individual based on their genetic makeup. For example, genetic analysis could show that a particular individual has a lower ability to metabolize certain micronutrients such as vitamin D and folate, so these can be recommended in diet as supplementation or replacement. This strategy can help in preventing the disorders that include diabetes, obesity, and cardiovascular disease by giving dietary recommendations more in line with the person's genetic profile (Garg et al. 2014).

Though genetic testing has immense benefits, major ethical concerns weigh against its widespread use, particularly concerning data privacy. Genetic data is very sensitive, and improper management may result in discrimination of people by insurance companies or employers on the basis of their genetic makeup. Several direct‐to‐consumer businesses maintain genetic data in their files, which places a vulnerability to possible security weaknesses or unauthorized entries. A stronger reason behind this is that the customer might not know what kind of implications the release of this genetic information may bring (Farhud and Yeganeh 2010).

Genes remain impossible to interpret in the right manner. There are known associations between certain genetic variants and nutritional effects. Others are unclear, or their studies are inadequate. This caution should be taken when creating diet programs tailored through genetic testing since dependency on partial genetic data may result in poor diets and choices (Dallio et al. 2021).

### 4.2 Artificial Intelligence and Machine Learning

The use of artificial intelligence and machine learning has been considered a revolution in personalized nutrition because it allows the analysis of vast databases to include genetic information, eating patterns, and health outcomes. These technologies can pinpoint intricate relationships between genes, lifestyle factors, and disease risks that are otherwise imperceptible to standard statistical techniques. For example, in terms of foreseeing how the general population might react to certain nutrients or dietary models, AI models may utilize research carried out from nutrigenomics, metabolomics, and microbiome studies (Ramos‐Lopez et al. 2017). These diets have enormous databases and could be assessed by using artificial intelligence or AI to determine the better diets that can reduce the risks of chronic diseases, such as diabetes and cardiovascular disease. AI‐driven algorithms can assess characteristics, such as genetic composition, metabolic reactions, and gut bacteria, to prescribe a particular diet for maximum health (Li et al. 2022a). This way, the doctor can provide even more dynamic and individualized nutritional counseling to a patient based on their individual biological information.

#### 4.2.1 Real‐World Applications of AI‐Driven Personalized Nutrition Platforms

Recently, many AI‐based personal nutrition companies surfaced that deliver personalized dietary recommendations for particular health data. Companies such as Day Two and Nutrigenomix rely on artificial intelligence to analyze genetic and microbiome data while providing people with personalized food programs according to their greatest health benefits. These portals suggest specific nutrients or foods that improve metabolic well‐being and reduce the incidence of disease using highly advanced algorithms that consider how a particular food works with any given person's biology (Kaur et al. 2018).

AI technologies can predict the response that a metabolic disorder patient, who may have diabetes or NAFLD, will have to the therapy offered through diet and can be applied in clinical settings. Systems of AI can change dietary suggestions in real time so that the blood glucose levels of the patients can become stabilized or prevent the buildup of liver fat by reviewing log data from dietary intake or continuous glucose monitoring (Dallio et al. 2021). This dynamic nutrition approach makes sure that therapies are always changed in keeping with the actual health conditions and, at the same time, are individualized.

### 4.3 Wearable Devices and Continuous Monitoring

Being that wearable technology and continuous monitoring tools would give real‐time information on how well a person's health indicators are, they have become essential to personalized nutrition. For instance, through continuous glucose monitoring devices, all‐day blood sugar levels are monitored, and therefore, how different foods and activities affect glucose metabolism is known. It helps diabetics to make proper decisions related to diet and insulin use by analyzing real‐time glucose readings. Normally, the additional Fitbit or Apple Watch information would have included heart rate, how much energy you are utilizing, and so on. Thus, by the combination of these measurements with dietary data, personalized nutrition platforms are able to provide recommendations that take into consideration not just your energy requirements but also your nutrient intake (Hossain, Wazed, Asha, Hossen, et al. 2025). A very interesting example is that a person who consumes plenty of physical exercise on a daily basis would require greater levels of protein and carbs in order to enhance muscle repair and energy levels. Such real‐time insights make diet adjustments dynamically and more precise.

#### 4.3.1 Integration of Real‐Time Data to Provide Dynamic, Tailored Recommendations

Personalized nutrition can make dynamic recommendations to the changing state of health by tapping real‐time streams of data coming from wearables, CGMs, and health applications. These systems have the potential to monitor a client's glucose levels, physical activity, and sleep patterns perpetually to allow for continuous real‐time revisions in the diet plan, thus maximizing health outcomes (Arshad et al. 2025). For example, if the CGM records a high level of blood sugar after a certain meal, the system may recommend decreasing the amount of carbs consumed or perhaps adjusting meals in a way that such surges do not happen again soon (Leshem et al. 2020). Additionally, smartwatches and fitness trackers, such as Fitbit or Apple Watch, record heart rate, energy expenditure, and physical activity, allowing personalized nutrition platforms to integrate these data with dietary intake and adjust recommendations dynamically. It highlighted AI's role in delivering real‐time, individualized dietary recommendations, particularly for chronic disease management, by analyzing complex health and dietary datasets (Karnika et al. 2025). These tools can also give quick feedback on whether the changes in nutrition are effective. For example, a person attempting to use a low‐carb diet can use a CGM to monitor what different foods do to their blood sugar control, thus aiding in their adjustment of the diet for better glycemic control. Similarly, tracking recovery parameters and calories burned means fitness trackers can enable understanding among the users of how well their diet will satisfy the needs of their physical activity goals. A higher degree of personalization and continuing input ensures that meal plans are effective and appropriate as their needs keep changing over time. Lastly, wearable technology and AI‐powered inventions are revolutionizing personal nutrition by providing timely, real‐time data for dynamic dietary advice alterations. These developments enhance the accuracy of nutrition interventions as well as their accessibility and utilization in persons who want to improve their health.

## 5 Conclusion

By integrating insights from nutrigenomics, nutrigenetics, microbiome research, and digital health technologies, it enables precise dietary recommendations that align with an individual's genetic, metabolic, and lifestyle profile. This approach has shown promise in improving metabolic flexibility, glycemic control, lipid metabolism, and overall disease prevention outcomes in obesity, type 2 diabetes mellitus, and cardiovascular disorders. However, the successful translation of personalized nutrition into clinical and public health practice requires overcoming several challenges, including high implementation costs, limited accessibility in low‐ and middle‐income countries, and ethical considerations surrounding data privacy and genetic testing. Future research should focus on establishing large‐scale, evidence‐based models that validate the clinical efficacy and cost‐effectiveness of personalized dietary interventions across diverse populations. Ultimately, the implementation of this integrative and evidence‐based approach could revolutionize preventative healthcare, reduce the global burden of non‐communicable diseases, and facilitate healthier, longer life through precision nutritional interventions.

## Author Contributions

**Muhammad Tayyab Arshad:** methodology (equal), writing – original draft (equal), writing – review and editing (equal). **M. K. M. Ali:** data curation (equal), validation (equal), writing – review and editing (equal). **Farhang Hameed Awlqadr:** investigation (equal), visualization (equal), writing – review and editing (equal). **Sammra Maqsood:** data curation (equal), methodology (equal), writing – review and editing (equal). **Ali Ikram:** formal analysis (equal), supervision (equal), writing – review and editing (equal). **Md. Sakhawot Hossain:** conceptualization (equal), data curation (equal), writing – review and editing (equal). **Muhammed Adem Abdullahi:** resources (equal), validation (equal), writing – review and editing (equal). **M. M. Rashed:** formal analysis (equal), investigation (equal), writing – review and editing (equal).

## Funding

This work was supported and funded by the Deanship of Scientific Research at Imam Mohammad Ibn Saud Islamic University (IMSIU) (grant number IMSIU‐DDRSP2502).

## Ethics Statement

The authors have nothing to report.

## Consent

The authors have nothing to report.

## Conflicts of Interest

The authors declare no conflicts of interest.

## Data Availability

The data supporting this study's findings are available from the corresponding author upon reasonable request.
